# Molecular imaging in nuclear cardiology: Pathways to individual precision medicine

**DOI:** 10.1007/s12350-020-02319-6

**Published:** 2020-09-06

**Authors:** A. Glasenapp, A. Hess, J. T. Thackeray

**Affiliations:** Department of Nuclear Medicine, Hannover Medical School, Translational Cardiovascular Molecular Imaging, Carl Neuberg Str 1, 30625 Hannover, Germany

**Keywords:** Positron emission tomography, Cardiovascular disease, Inflammation, Fibrosis, Sympathetic nervous system

## Abstract

Growth of molecular imaging bears potential to transform nuclear cardiology from a primarily diagnostic method to a precision medicine tool. Molecular targets amenable for imaging and therapeutic intervention are particularly promising to facilitate risk stratification, patient selection and exquisite guidance of novel therapies, and interrogation of systems-based interorgan communication. Non-invasive visualization of pathobiology provides valuable insights into the progression of disease and response to treatment. Specifically, inflammation, fibrosis, and neurohormonal signaling, central to the progression of cardiovascular disease and emerging therapeutic strategies, have been investigated by molecular imaging. As the number of radioligands grows, careful investigation of the binding properties and added-value of imaging should be prioritized to identify high-potential probes and facilitate translation to clinical applications. In this review, we discuss the current state of molecular imaging in cardiovascular medicine, and the challenges and opportunities ahead for cardiovascular molecular imaging to navigate the path from diagnosis to prognosis to personalized medicine.

**Electronic supplementary material:**

The online version of this article (10.1007/s12350-020-02319-6) contains supplementary material, which is available to authorized users.

**Electronic supplementary material:**

The online version of this article (10.1007/s12350-020-02319-6) contains supplementary material, which is available to authorized users.

## Introduction

**Figure 1:**
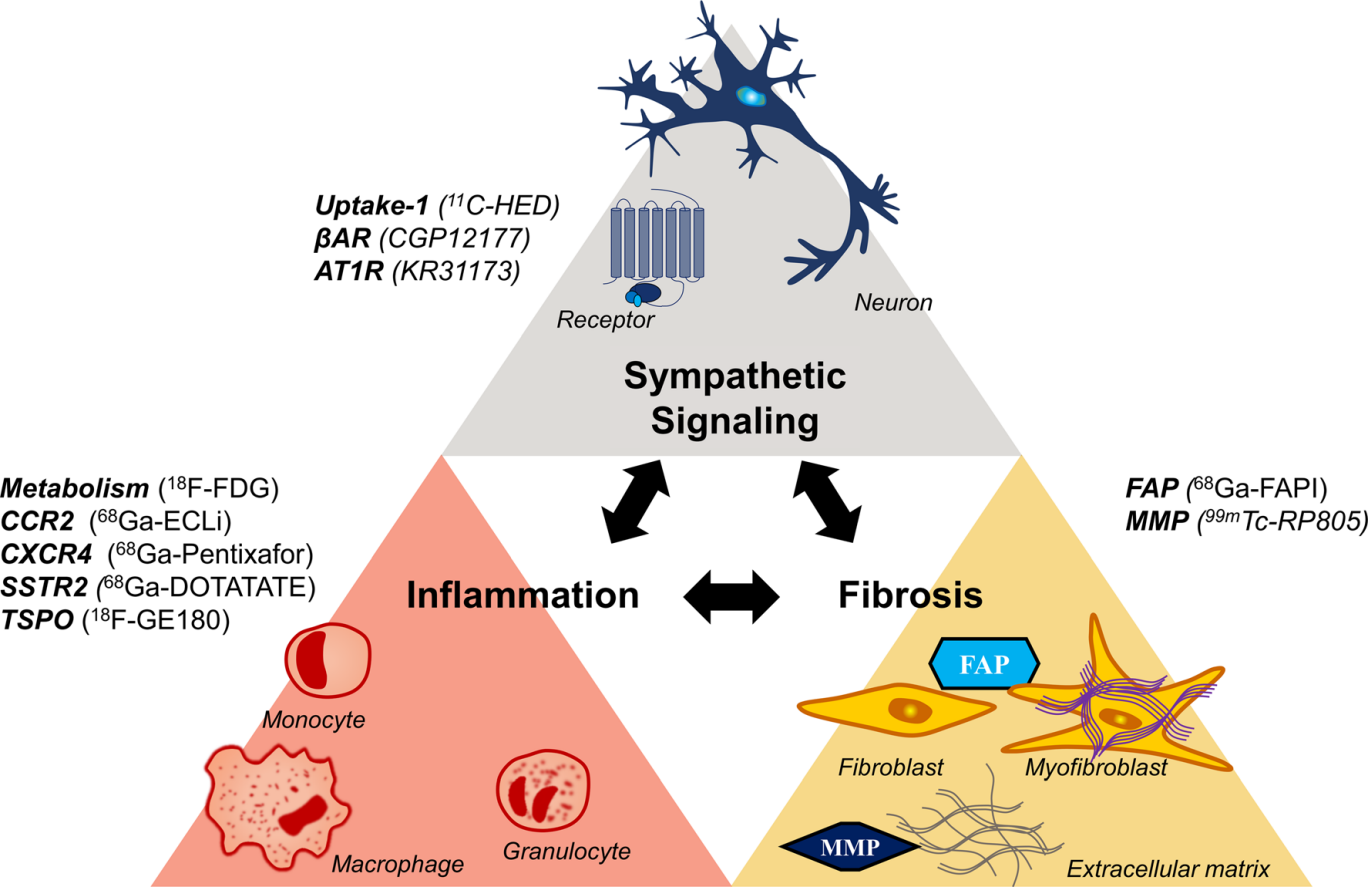
Overview of cardiovascular molecular imaging. Pathogenetic processes targeted by current radiopharmaceuticals include inflammatory leukocytes, fibroblasts, and proteases involved in matrix reorganization and sympathetic neuronal signaling. Each pathway is thought to influence the others by means of cytokines or signal transduction cascades

As cardiovascular precision medicine embraces molecular-targeted therapies, the identification of at-risk and likely-to-respond patients takes on greater importance. Imaging to non-invasively quantify these molecular targets can provide incremental value in selecting appropriate patient populations for selective and expensive therapies. Accordingly, nuclear cardiology finds itself at a critical junction, where the pathway demarcated by image-guided oncology may direct the future of cardiovascular molecular imaging. Conventional nuclear cardiology assesses myocardial perfusion, viability, function, and scar—i.e., measurements of disease severity after initial insult.^1^ However, these measures are generally observational and provide only limited opportunity for novel intervention, particularly at the molecular level. Accordingly, the development of new molecular-targeted imaging probes enables imaging at earlier stage of disease, building toward patient risk stratification, therapeutic guidance, and systems-based evaluations. To this end, the pathophysiological mechanisms of inflammation, fibrosis, and neurohormonal signaling have come to the forefront of molecular imaging in nuclear cardiology (Figure 1).

### Inflammation

Inflammation critically contributes to development and progression of cardiovascular disease. After ischemic injury, cardiomyocyte death initiates release of pro-inflammatory factors, followed by leukocyte infiltration, remodeling, and repair. High circulating blood leukocytes are associated with higher mortality and adverse cardiac events among patients.^2^ Serum-based biomarkers, such as high sensitivity C-reactive protein, while widely used, are a crude indicator of local tissue inflammation, and accurate measurement of the injury microenvironment typically requires invasive biopsy. Molecular imaging enables a non-invasive ‘virtual biopsy,’ providing added-value in diagnosis and prognosis. Moreover, precise molecular therapies are emerging (e.g., antibodies and small peptides) which target specific components of the inflammatory pathway and bear potential to identify early pathological mechanisms for treatment to improve outcome.^1^ Early local inflammation after myocardial infarction (MI) in mice predicts functional outcome and provides guidance for precisely targeted and timed intervention.^3^

**Figure 2:**
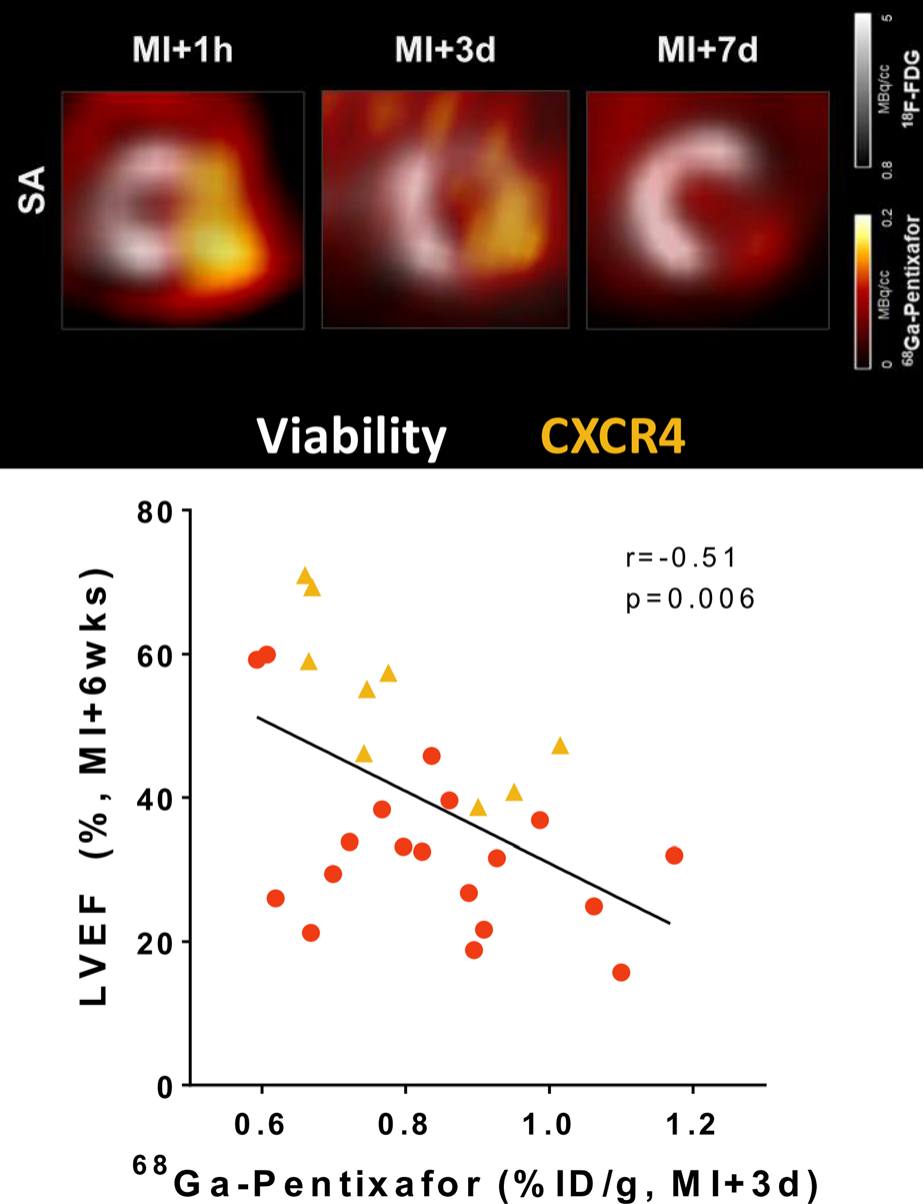
Molecular imaging of chemokine receptor CXCR4 after myocardial infarction. Transient upregulation of CXCR4 PET signal (colourscale) in the non-viable infarct zone (FDG, greyscale) at 1 hour and 3 days after coronary artery occlusion declines by 7 days in mice. The PET signal at 3 days predicts left ventricle ejection fraction (LVEF) 6 weeks later. Prepared using data from Hess et al. *Eur Heart J* 2020^3^

Contrary to the robust local inflammatory response after MI, non-ischemic cardiac diseases are characterized by diffuse myocardial inflammation, a greater challenge for imaging. The inflammatory response can be triggered by mechanical strain, neurohormonal activation, oxidative stress, fibrosis, and/or modest cardiomyocyte necrosis.^4^ Treatments typically minimize symptoms and improve quality of life, whereby blockbuster drugs delay or lessen remodeling but cannot avert disease progression.^5^ Early inflammation provides a therapeutic avenue which may complement conventional therapy, such that precise characterization of the temporal and spatial inflammatory cell invasion can predict subsequent outcome (Figure 2). The presence of inflammation in atherosclerosis prior to coronary artery disease predicts future adverse cardiac events.^6^

As such, a number of molecular imaging agents have been explored for characterization of cardiac inflammation (Table 1). While most clinical experience relies on ^18^F-fluorodeoxyglucose, novel molecular radioligands portend the opportunity to distinguish specific cellular components of the inflammatory response.^7^,^8^ These radioligands are most effective when coupled to specific therapies via the same molecular target, as for chemokine receptors.^3^,^9^

**Table 1:** Molecular imaging radioligands for cardiovascular inflammation

Tracer	Molecular target	Cells	Stage of research
^11^C-Methionine	Amino acid uptake	Activated macrophages	Preclinical/clinical
^18^F-FDG	Glucose transporter 4	Activated macrophages, cardiomyocytes	Clinical
^18^F-GE180	Translocator protein (TSPO)	Activated macrophages, microglia	Clinical
^18^F-Mannose	Mannose receptor	Reparative macrophages	Preclinical
^68^Ga-DOTA-ECL1i	Chemokine receptor CCR2	Pro-inflammatory leukocytes (Ly6C^high^ monocytes)	Preclinical
^68^Ga-DOTATATE	Somatostatin receptor type 2 (SSTR2)	Activated macrophages	Preclinical/clinical
^68^Ga-Pentixafor	Chemokine receptor CXCR4	Leukocytes	Preclinical/clinical

### Fibrosis

Myocardial fibrosis is a common endpoint of cardiovascular disease, characterized by resident cardiac fibroblast transdifferentiation and activation, which produce fibrillary collagen and reorganize extracellular matrix. Reparative or replacement fibrosis after ischemic injury culminates in scar formation and stabilization of the infarct.^10^ Reactive fibrosis is stimulated by local myocyte death, mechanical stimulus, or neurohormonal activation, leading to myofibroblast transdifferentiation and interstitial collagen deposition.^11^ The extended duration of the pathologic impetus, e.g., pressure or volume overload, cardiomyopathy, cardiotoxicity, infection, and metabolic stress, evokes prolonged myofibroblast activation, and progressive fibrogenesis over time.^12^

Non-invasive characterization of fibrosis typically relies on estimation of ventricle stiffness and filling via echocardiography or characterization of tissue differences via cardiac magnetic resonance imaging.^13^,^14^ Prolonged T1 relaxation time on cardiac magnetic resonance imaging correlates to diffuse cardiac fibrosis in biopsy samples,^15^ suggesting the possibility to non-invasively characterize fibrotic burden in heart failure patients. But these measurements target the result of fibroblast activation, mature scar, or interstitial collagen late in disease progression. Accordingly, biomarkers of fibroblast activation early in pathogenesis are desirable. The fibroblast activation protein (FAP) is highly expressed by activated (myo)fibroblasts and is upregulated in response to ischemic and non-ischemic cardiomyopathy.^16^

To date, therapies to directly mitigate cardiac fibrosis are lacking, though novel strategies including gene transfer to reprogram cardiac fibroblasts^17^ or chimeric antigen T cells directed against FAP^18^ have shown promise in animal studies. Notably, conventional clinical management, including blockbuster drugs, slows the fibrotic mechanisms in hypertensive and heart failure patients, though the mechanism remains unclear. As such, visualization and quantification of early fibroblast activity provide insights into the pathology which may aid in drug development and optimization.

**Figure 3:**
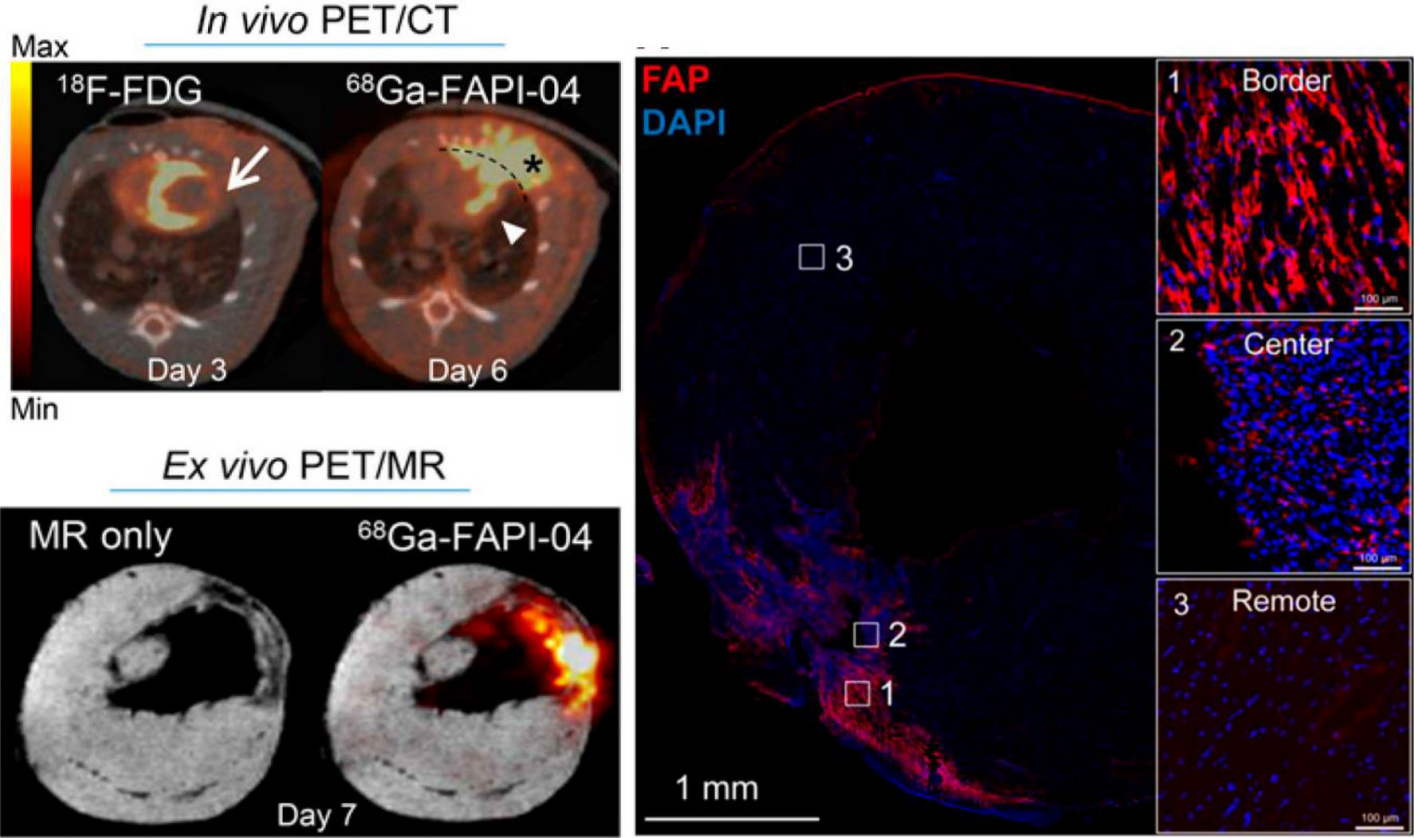
Visualization of fibroblast activation after myocardial infarction. Increased fibroblast activation protein (FAP) expression identified by ^68^Ga-FAPI-04 signal on PET-CT and ex vivo PET-MR in rats. Immunohistology confirmed high FAP expression in infarct border zone by myofibroblasts. Reproduced with permission from Varasteh et al. *J Nucl Med*. 2019 19

The expansion of imaging approaches for early indicators of fibrosis has stimulated interest in applying FAP-targeted imaging for cardiovascular disease. After permanent coronary artery ligation in rats, ^68^Ga-labeled FAP inhibitor accumulated in the infarct territory at 6d after injury, receding to baseline subsequently. Signal specificity was confirmed by blocking and immunofluorescence staining. The density of FAP-positive fibroblasts was significantly higher in the infarct border zone compared to center or remote myocardium,^19^ suggesting the visualization of infarct expansion (Figure 3). Further research into prognosis and therapy response with FAP imaging and application in non-focal fibrotic disease is warranted.

### Neurohormonal Signaling

The sympathetic nervous system is the primary extrinsic control of heart rate and contractility. Heightened sympathetic signaling compensates for the failing heart, leading to downregulation of adrenoceptors and excitation–contraction uncoupling. Beta-blocker therapy initially inhibits the over-stimulation of adrenoceptors, re-establishing homeostasis in autonomic regulation of contractile function.^20^ High sensitivity of sympathetic neurons to ischemia leads to selective dysinnervation of the heart after MI, which has been implicated as a substrate of ventricular arrhythmia and sudden cardiac arrest.^21^,^22^

**Figure 4:**
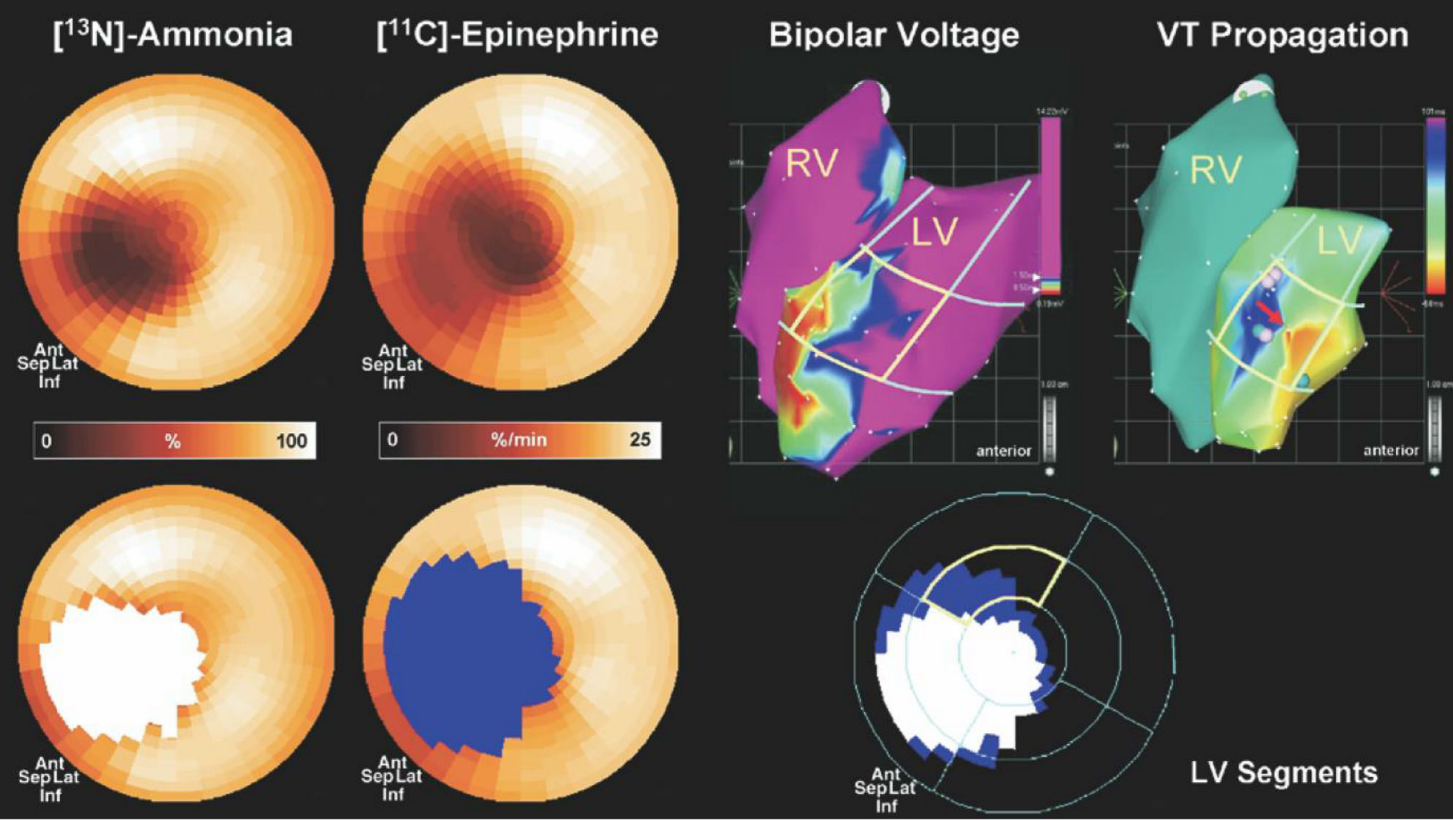
Imaging of cardiac sympathetic denervation identifies substrate of arrhythmia. Innervation defect defined by ^11^C-epinephrine exceeds the perfusion defect and colocalized to site of initiation of ventricular fibrillation on electrophysiology study after myocardial infarction in pigs. Reproduced with permission from Sasano et al. *J Am Coll Cardiol*. 2008^21^

Imaging of the cardiac sympathetic nervous system has been pursued over the last three decades, but the impact on clinical care has been underwhelming. The principal limitation of innervation imaging lies with the radiotracers themselves, which are largely subject to variable permutations of neuronal reuptake, vesicular packaging, active synaptic release, passive diffusion to the synaptic cleft, and metabolic degradation. Despite evidence supporting the role of denervated myocardium in sudden cardiac arrest and heart failure progression (Figure 4), the limitations of quantification and tracer availability have prevented translation. Some of this hesitancy relates to the cost-effectiveness of imaging vs the fairly inexpensive cost of anti-adrenergic drugs. Newer compounds with favorable labeling and kinetics and targeting other signaling components such as angiotensin II type 1 receptors have been proposed,^23^,^24^ but have not yet seen widespread clinical application. Whether sympathetic neuronal imaging can be buoyed by these developments and connections to device therapy will ultimately determine its future.

### Challenges and Opportunities

The expansion of the molecular imaging radiotracer arsenal provides a number of opportunities for research and patient management (Table 2). When these agents target pathogenetic mechanisms early in disease progression, they can facilitate risk stratification based on the expression pattern of inflammation, fibrosis, or sympathetic neuronal dysfunction at the site of injury. This approach allows regional organ interrogation at the site of injury and offers unique insight into pathobiology. Importantly, shared targets for imaging and therapeutic agents offer the potential to monitor early mechanisms of pathogenesis and direct clinical interventions toward patients at highest risk. Suitable patients and the optimal time point for treatment or intervention could be identified based on the temporal imaging signal.

**Table 2:** Challenges and opportunities for cardiovascular molecular imaging

Challenge	Opportunity
Tracer sensitivity	Blocking studies for target specificity
	Species differences in targets and affinity
	Focal vs diffuse target expression
	Test–retest reproducibility of signal
Prognostic value	Quantitative tracer signal in disease models
	Timecourse evaluation of disease-based signal (optimal timepoint)
	Outcomes-based data to relate early signal to late function
Therapeutic response	Tracer sensitivity to therapeutic response
	Timecourse evaluation of therapeutic response
Systems interaction	Whole body analysis
Pathway interface	Multi-tracer studies and timecourse evaluation

To this end, targeted imaging and treatment of inflammatory and fibrotic mechanisms provide the opportunity to interrogate the intersection of these processes, which can further refine treatment strategies to benefit the individual patient. Moreover, the growing capacity to acquire images beyond the target organ, either through multiple bed positions or total-body PET, enables systems-based analysis, offering unique insights into the interaction of different organ systems. As such, cardiovascular molecular imaging can define the pathway to precision patient management, giving critical insights into disease processes, early prognosis, and response to therapy that can ultimately lead the right patient to the right therapy on the appropriate schedule.

## Electronic supplementary material

Below is the link to the electronic supplementary material.

Supplementary material 1 (PPDX 2043 kb)

Supplementary material 2 (M4A 3594 kb)

Supplementary material 3 (DOCX 12 kb)

## Abbreviations

FAP: Fibroblast activation protein
MI: Myocardial infarction
