# Supplementary material for: Molecular imaging in nuclear cardiology: Pathways to individual precision medicine
Source: J Nucl Cardiol. 2020 Sep 6;27(6):2195–201. doi: 10.1007/s12350-020-02319-6 (PMC7749093; doi:10.1007/s12350-020-02319-6)
Supplement: Supplementary file 1 — Supplementary material 1 (PPDX 2043 kb) [file 12350_2020_2319_MOESM1_ESM.pptx]

## Slide 1
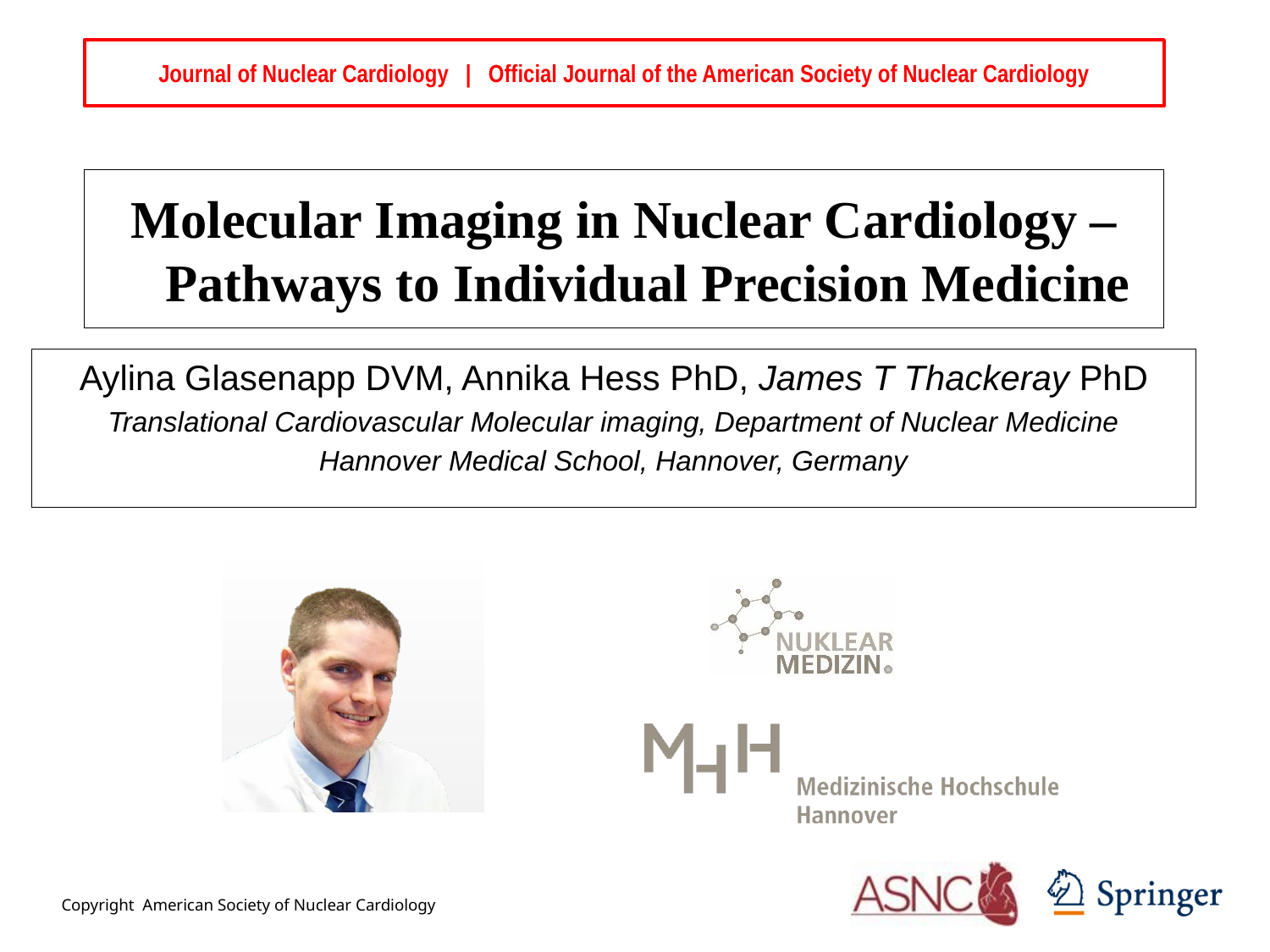

Journal of Nuclear Cardiology | Official Journal of the American Society of Nuclear Cardiology
# Molecular Imaging in Nuclear Cardiology – Pathways to Individual Precision Medicine
Aylina Glasenapp DVM, Annika Hess PhD, James T Thackeray PhD
Translational Cardiovascular Molecular imaging, Department of Nuclear Medicine
Hannover Medical School, Hannover, Germany
Copyright American Society of Nuclear Cardiology

## Slide 2
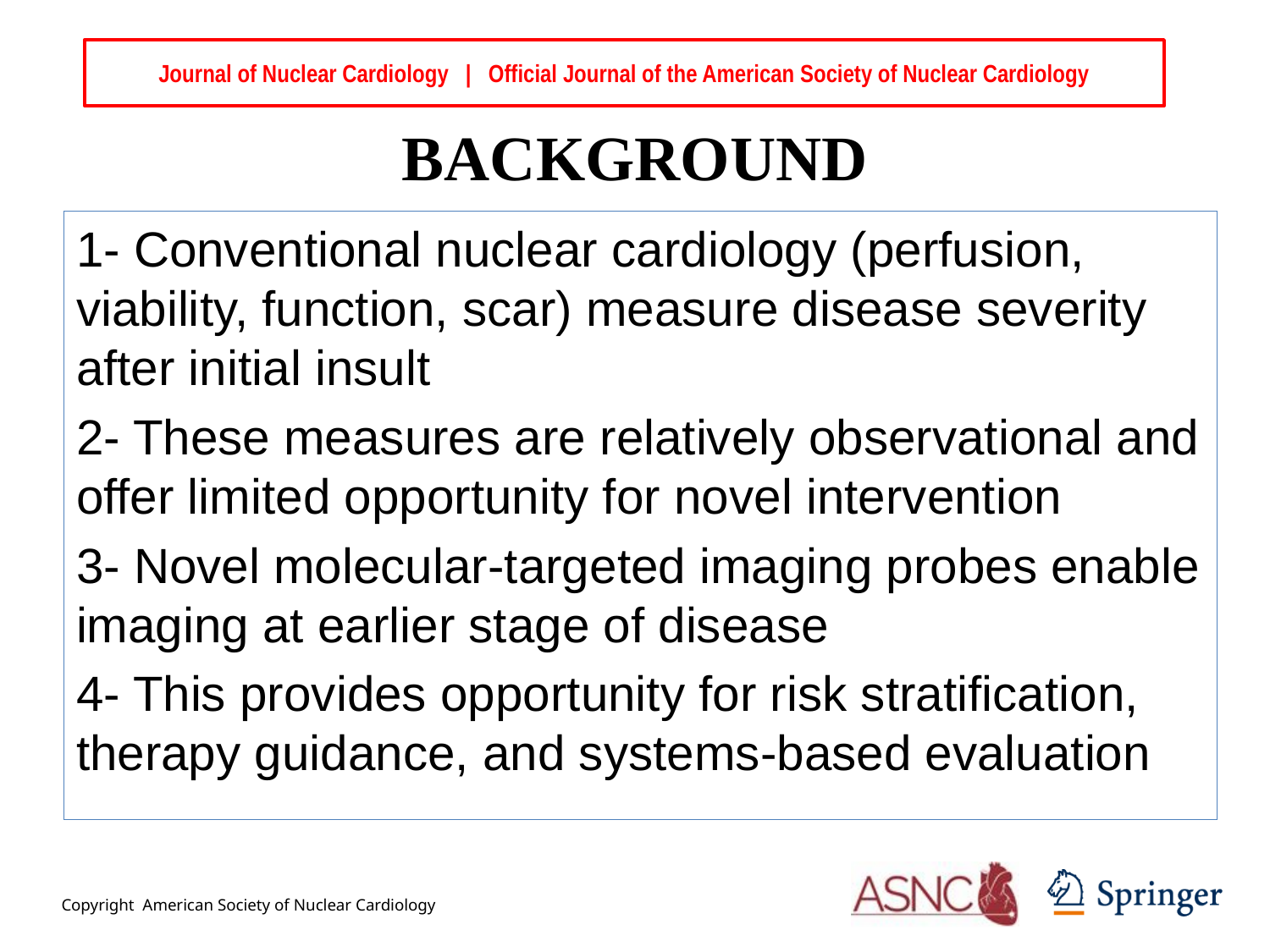

Journal of Nuclear Cardiology | Official Journal of the American Society of Nuclear Cardiology
# BACKGROUND
1- Conventional nuclear cardiology (perfusion, viability, function, scar) measure disease severity after initial insult
2- These measures are relatively observational and offer limited opportunity for novel intervention
3- Novel molecular-targeted imaging probes enable imaging at earlier stage of disease
4- This provides opportunity for risk stratification, therapy guidance, and systems-based evaluation
Copyright American Society of Nuclear Cardiology

## Slide 3
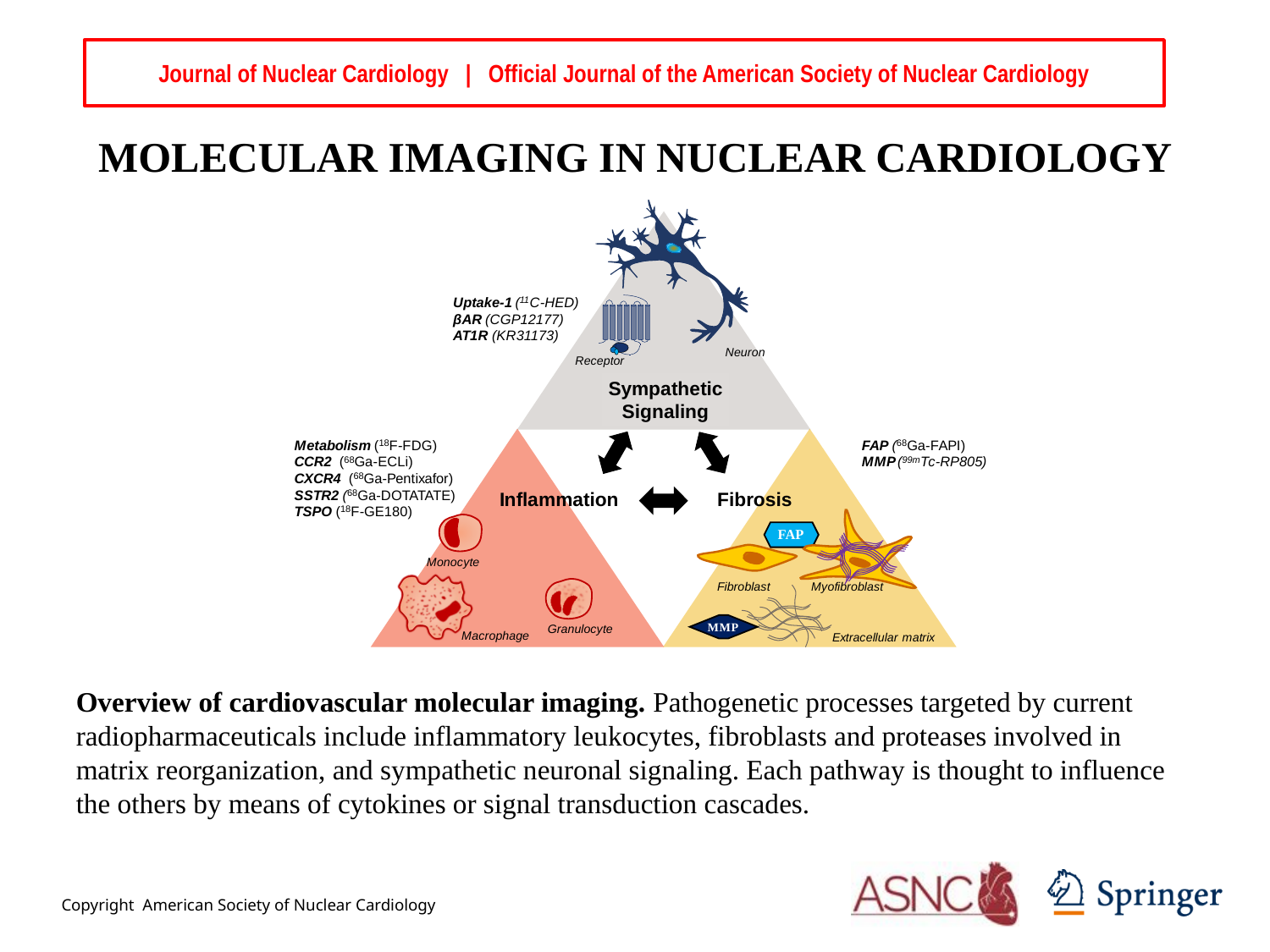

Journal of Nuclear Cardiology | Official Journal of the American Society of Nuclear Cardiology
# Molecular Imaging in Nuclear Cardiology
Overview of cardiovascular molecular imaging. Pathogenetic processes targeted by current radiopharmaceuticals include inflammatory leukocytes, fibroblasts and proteases involved in matrix reorganization, and sympathetic neuronal signaling. Each pathway is thought to influence the others by means of cytokines or signal transduction cascades.
Copyright American Society of Nuclear Cardiology

## Slide 4
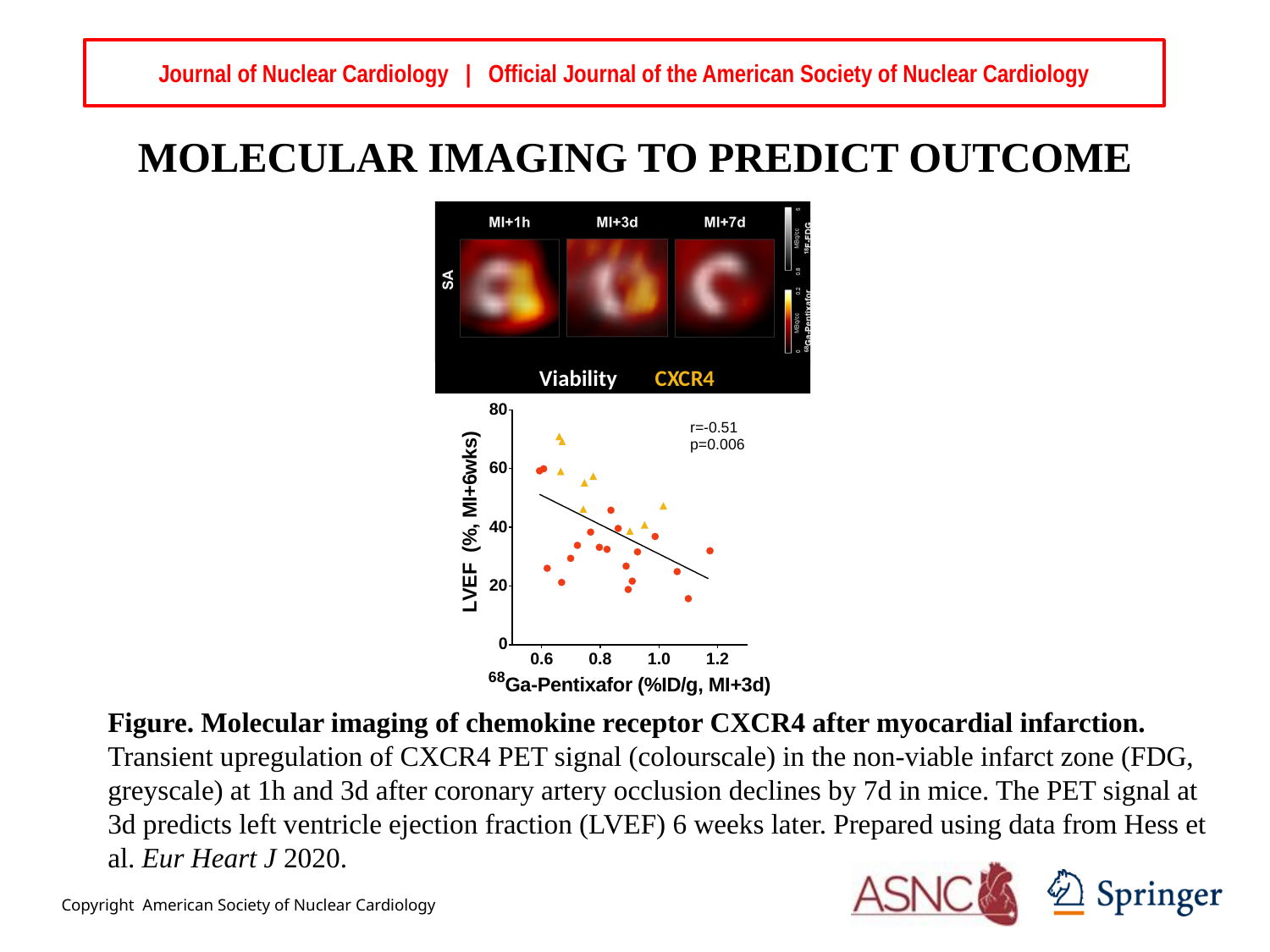

Journal of Nuclear Cardiology | Official Journal of the American Society of Nuclear Cardiology
# Molecular Imaging to Predict Outcome
Figure. Molecular imaging of chemokine receptor CXCR4 after myocardial infarction. Transient upregulation of CXCR4 PET signal (colourscale) in the non-viable infarct zone (FDG, greyscale) at 1h and 3d after coronary artery occlusion declines by 7d in mice. The PET signal at 3d predicts left ventricle ejection fraction (LVEF) 6 weeks later. Prepared using data from Hess et al. Eur Heart J 2020.
Copyright American Society of Nuclear Cardiology

## Slide 5
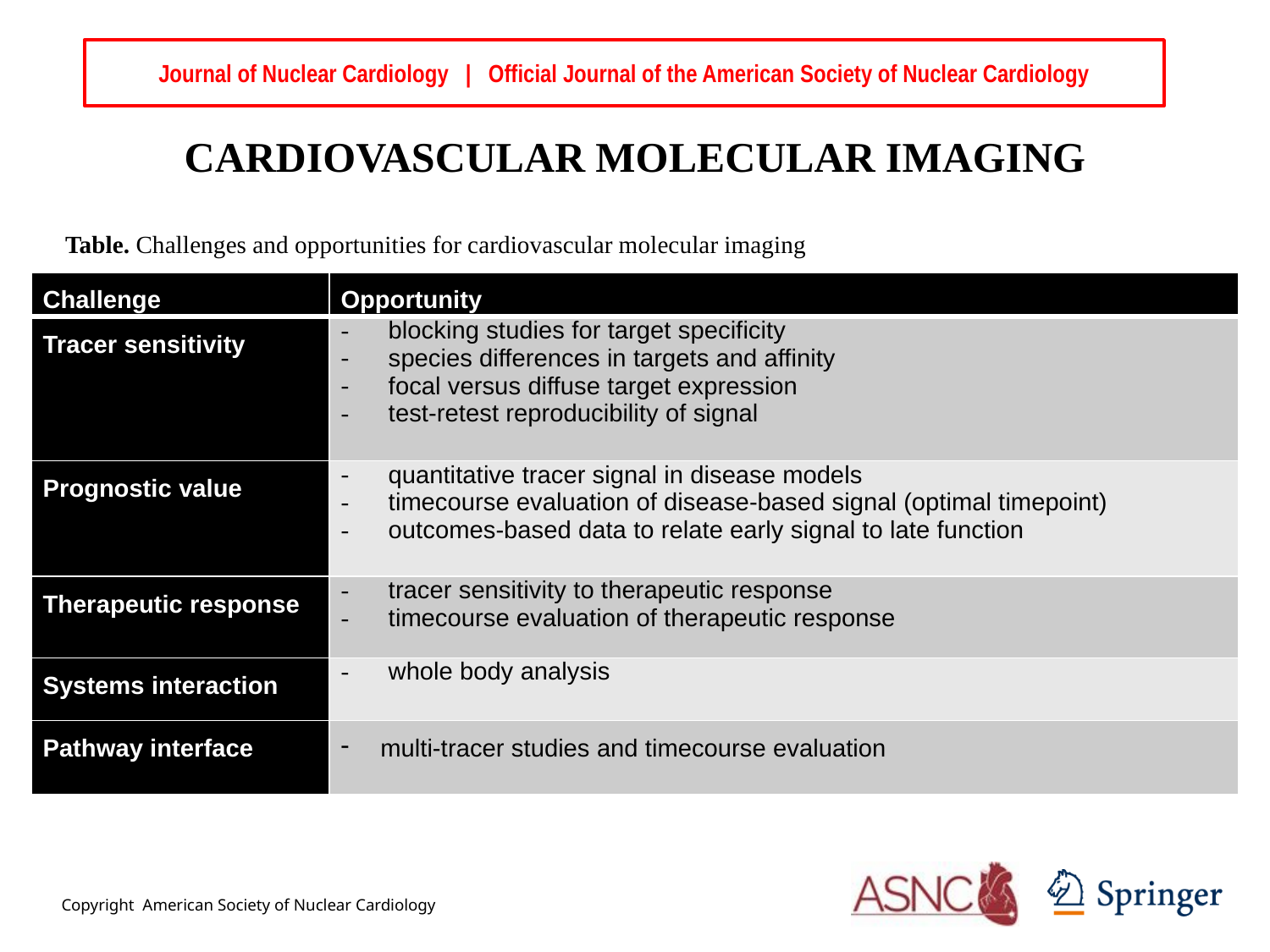

Journal of Nuclear Cardiology | Official Journal of the American Society of Nuclear Cardiology
# Cardiovascular Molecular Imaging
Table. Challenges and opportunities for cardiovascular molecular imaging
| Challenge | Opportunity |
| --- | --- |
| Tracer sensitivity | blocking studies for target specificity species differences in targets and affinity focal versus diffuse target expression test-retest reproducibility of signal |
| Prognostic value | quantitative tracer signal in disease models timecourse evaluation of disease-based signal (optimal timepoint) outcomes-based data to relate early signal to late function |
| Therapeutic response | tracer sensitivity to therapeutic response timecourse evaluation of therapeutic response |
| Systems interaction | whole body analysis |
| Pathway interface | multi-tracer studies and timecourse evaluation |
Copyright American Society of Nuclear Cardiology

## Slide 6
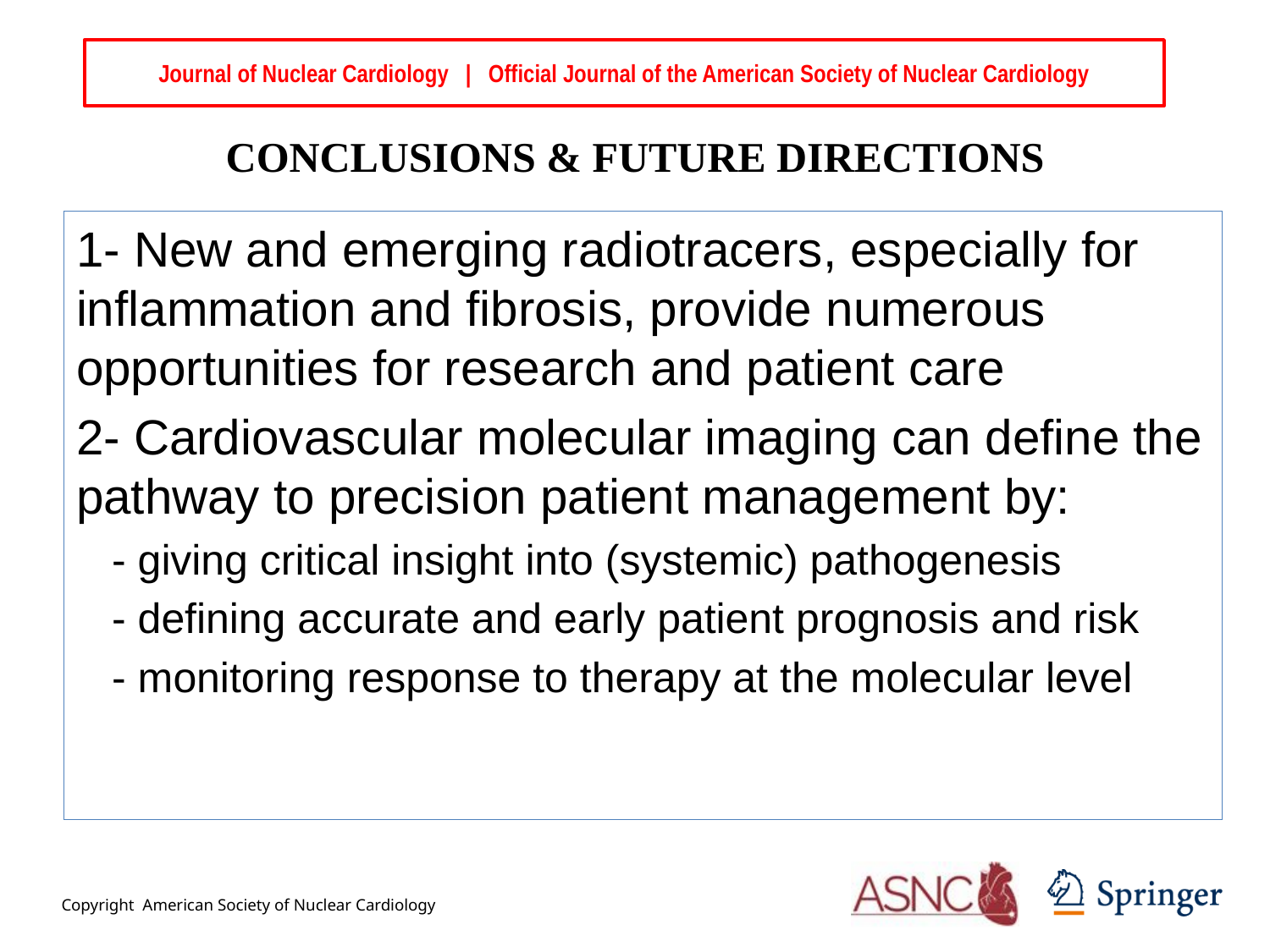

Journal of Nuclear Cardiology | Official Journal of the American Society of Nuclear Cardiology
# CONCLUSIONS & FUTURE DIRECTIONS
1- New and emerging radiotracers, especially for inflammation and fibrosis, provide numerous opportunities for research and patient care
2- Cardiovascular molecular imaging can define the pathway to precision patient management by:
 - giving critical insight into (systemic) pathogenesis
 - defining accurate and early patient prognosis and risk
 - monitoring response to therapy at the molecular level
Copyright American Society of Nuclear Cardiology
