# Supplementary material for: Molecular imaging in nuclear cardiology: Pathways to individual precision medicine
Source: J Nucl Cardiol. 2020 Sep 6;27(6):2195–201. doi: 10.1007/s12350-020-02319-6 (PMC7749093; doi:10.1007/s12350-020-02319-6)
Supplement: Supplementary file 3 — Supplementary material 3 (DOCX 12 kb) [file 12350_2020_2319_MOESM3_ESM.docx]

Navigating the path to personalized medicine guided by molecular imaging of inflammation, fibrosis and neurohormonal signaling. Emerging opportunities in cardiovascular imaging.
